# The Way Humans Behave Modulates the Emotional State of Piglets

**DOI:** 10.1371/journal.pone.0133408

**Published:** 2015-08-05

**Authors:** Sophie Brajon, Jean-Paul Laforest, Océane Schmitt, Nicolas Devillers

**Affiliations:** 1 Agriculture and Agri-Food Canada, Dairy and Swine Research and Development Centre, 2000 College Street, Sherbrooke, Quebec, J1M 0C8, Canada; 2 Université Laval, Department of Animal Science, 2325 Rue de l’Université, Quebec city, Quebec, G1V 0A6, Canada; Université Pierre et Marie Curie, FRANCE

## Abstract

The emotional state can influence decision-making under ambiguity. Cognitive bias tests (CBT) proved to be a promising indicator of the affective valence of animals in a context of farm animal welfare. Although it is well-known that humans can influence the intensity of fear and reactions of animals, research on cognitive bias often focusses on housing and management conditions and neglects the role of humans on emotional states of animals. The present study aimed at investigating whether humans can modulate the emotional state of weaned piglets. Fifty-four piglets received a chronic experience with humans: gentle (GEN), rough (ROU) or minimal contact (MIN). Simultaneously, they were individually trained on a go/no-go task to discriminate a positive auditory cue, associated with food reward in a trough, from a negative one, associated with punishments (e.g. water spray). Independently of the treatment (*P* = 0.82), 59% of piglets completed the training. Successfully trained piglets were then subjected to CBT, including ambiguous cues in presence or absence of a human observer. As hypothesized, GEN piglets showed a positive judgement bias, as shown by their higher percentage of go responses following an ambiguous cue compared to ROU (*P* = 0.03) and MIN (*P* = 0.02) piglets, whereas ROU and MIN piglets did not differ (*P* > 0.10). The presence of an observer during CBT did not modulate the percentage of go responses following an ambiguous cue (*P* > 0.10). However, regardless of the treatment, piglets spent less time in contact with the trough following positive cues during CBT in which the observer was present than absent (*P* < 0.0001). This study originally demonstrates that the nature of a chronic experience with humans can induce a judgement bias indicating that the emotional state of farm animals such as piglets can be affected by the way humans interact with them.

## Introduction

Domestic animals are considered to be sentient and endowed with cognitive and emotional abilities. The fact that domestic animals can experience emotional states has resulted in developing methods for welfare assessment and monitoring. However, animal welfare legislation often focusses on housing and management conditions and less on how people behave and interact with them. Yet humans can influence reactions of animals towards them and affect their behaviour and physiology [1].

Emotions can influence cognitive processes including attention, learning, memory and judgement. Emotions refer to an intense but short-lived pattern of affective processes linked to a stimulus and are associated with physiological and behavioural changes [2, 3]. They arise in relevant situations for individuals and influence the appraisal of stimuli and the decision-making in order to optimise survival and reproductive success [4, 5]. On the other hand, affective states may be rather viewed as long-lived mood states that can vary both in terms of valence (pleasantness / unpleasantness) and intensity but the terms “emotions” and “affective states” are often used interchangeably [3]. The term “cognitive bias” has been proposed to label the effects of long-term affective states (i.e. mood) on cognitive functions [6], although it is sometimes used to label acute changes in affective states (i.e. emotions) [7]. Research on cognitive bias provides a non-invasive and promising tool for assessing welfare of domestic and captive animals. Affective states influence memory retrieval by inducing preferential implicit recall of negative information in depressed people and by inducing preferential implicit recall of positive information in happier people [8]. Likewise, positive affective state (“optimism”) increases the likelihood to expect a positive outcome (i.e. a reward) from an ambiguous situation whereas negative affective state (“pessimism”) increases the likelihood to expect a negative outcome (i.e. no reward, smaller reward or punishment) [3, 6]. Particularly, the study of judgement bias, or “risk taking” under uncertainty, and how affective state modulates the way individuals interpret information often involves various cognitive processes such as attention, memory and perception [3]. It has gained increasing attention recently and has been studied in a wide range of species from honeybees [7] to psittacines [9], rats [10, 11] and humans [12]. In most cases, individuals were trained to discriminate between positive and negative cues by an operant conditioning and then, their behavioural response was tested with unknown ambiguous cues.

Although there is a growing interest to use the judgement bias test to assess animal welfare [4], few studies have applied this test to evaluate the role of humans on moods. Yet the way that humans behave with domestic animals may influence their emotional state. For example, in a study in horses [13], animals trained using negative reinforcements (i.e. gentle pressure on the halter, gently shaking or pulling the lead, gentle pressure on the flank of the horse using a stick) developed more optimistic bias than horses trained using positive reinforcements (i.e. using a clicker followed by a food reward, “clicker method”, Kurland (1999) cited by Briefer Freymond et al. [13]). The authors suggested that this optimistic bias may be induced by the release from negative emotions experienced with the trainer during training. In contrast, animals may also associate humans with positive emotions and suffer from the separation with a familiar gentle human. For instance, it has been demonstrated that dogs showing separation-related behaviour in the absence of their owner exhibited a pessimistic judgement bias compared to other dogs [14]. However, to our knowledge, the influence of positive compared to negative interactions with humans has not been investigated in farm animals. Since the human-animal relationship on farms is a central question in terms of animal welfare [15], investigation of emotional states of farm animals following a previous experience with humans should be of great interest. Pigs are gregarious animals naturally seeking to interact with humans but, with experience, they can associate humans with positive or negative properties and remember their relationship with these particular humans [16–20]. The long shared history with humans [21] make pigs relevant models for the study of inter-specific relationships and emotions as expressed by a domestic species. Moreover, the cognitive bias test has been used successfully in pigs and helped demonstrating that environmental enrichment induces optimistic judgement bias [22] whereas stocking density or repeated social isolation and restraint did not generate judgement biases in pigs [23–25].

The aim of the present study was to investigate whether a previous experience with humans could affect or improve emotional state of pigs by using the judgement bias paradigm. It was also investigated whether or not the presence of a familiar handler could induce an affective arousal and influence the behaviour of the piglets during cognitive bias test. It was hypothesised that a positive experience with humans would induce an optimistic-like judgement bias whereas a negative experience with humans would induce a pessimistic-like judgements bias. It was also hypothesised that the presence of the human associated with the negative or the positive experience affects the performance of piglets during cognitive bias tests.

## Material and Methods

### Ethical statement

Animals were cared for according to the recommendations in the Canadian Council on Animal Care guidelines on the care and use of farm animals in research, teaching and testing [26] and the experimental protocol was approved by the Institutional Animal Care Committee of the Dairy and Swine Research & Development Centre (Sherbrooke, QC, Canada) (authorisation #437).

### Animals and housing conditions

A total of 54 weaned piglets from nine litters ((Yorkshire x Landrace) x Duroc), born at the experimental piggery of the Dairy and Swine Research & Development Centre (Sherbrooke, QC, Canada), were allocated to two blocks of nine groups of three piglets. The 18 groups (6 piglets/treatment) were weaned at 21 ± 2 days of age and housed in rooms containing three pens of 3.46 m^2^ made of plastic-coated expanded metal flooring. Rooms were supplemented with artificial light between 0700 and 1900 hours. Temperature was maintained between 22°C and 25°C and water was supplied *ad libitum*. Each group was composed of subjects of both genders from three different litters, with a difference of at least 1 kg weaning weight between the medium and both the lightest and the heaviest piglets. Teeth were not clipped or ground, tails were not docked and males were not castrated. Following the weaning, piggery staff was not allowed to enter the pens or to handle piglets and could only visually check their health status once a day and fill feeders.

During the first two weeks following weaning, commercial feed was supplied *ad libitum* to allow piglets to become familiar with solid food. From the third week post-weaning, piglets were rationed at 90% from Monday to Friday with 1/3 of the daily ration on the morning and 2/3 on the afternoon in order to increase their motivation for food reward during behavioural tests. Piglets were still fed *ad libitum* during weekends (from Friday afternoon to Sunday afternoon) to evaluate the quantity of food spontaneously ingested and to calculate the amount of food to be given to each group for the following week. Ration was eventually adjusted and increased if piglets had eaten all the afternoon meal within 30 min or decreased if it was not finished by the following morning. At the end of experiments, subjects were returned to the current management of farm production.

### Experimental design

Piglets were simultaneously subjected to one of the three treatments relative to different types of experience with humans in their home pen and trained to a go/no-go task in a testing arena. Since moving animals from the home pen to the testing arena involved interactions between piglets and humans, it was decided to match the moving procedure to the handling treatment. Treatment sessions started on the third day post-weaning and training sessions started on the tenth day post-weaning. Piglets were trained to the go/no-go task until they were able to discriminate positive from negative cues. Once the training was completed, piglets were tested in a cognitive bias test (CBT) which involved the addition of three new ambiguous cues comprised between positive and negative trained cues.

### Treatments

Treatments were given from the third day post-weaning until the end of experiment. Groups of piglets received either gentle contact (N = 18 piglets, GEN), rough contact (N = 18 piglets, ROU) or minimal contact (N = 18 piglets, MIN) in their home pen. Each room contained piglets of a same treatment only.

*Gentle contact* (GEN) aimed at gradually habituating piglets to the human presence, movements and contact from the third to the tenth day post-weaning (four sessions of 5 min/day, 5 d/week), and then to maintain this relationship during the rest of the experiment (two sessions of 2 min/day, 5 d/week). During the first sessions, the handler was crouched and talked softly to piglets, let them explore her and gradually tried to stroke them. In following sessions, the handler also stood up and walked quietly and smoothly in the pen. Piglets were considered as habituated when the handler was able to change her posture, walk quietly, talk and stroke them without provoking any escape. On the two last days before beginning the training for the go/no-go test, piglets received four sessions of 10 min habituation to the trolley with the same familiar handler. A trolley with a ramp was introduced into the home pen and the handler attracted piglets inside using food rewards (raisins). Four sessions were sufficient for piglets to go into and out of the trolley voluntarily.

*Rough contact* (ROU) aimed at maintaining fear of humans by piglets throughout the experiment. The number and duration of sessions was kept minimal to maintain fearfulness and avoid habituation. Twice per week during 2 min, the handler pursued piglets, hit walls with hands and floor with feet, talked loudly and abruptly and applied one of the following sudden and unpredictable rough interactions to each piglet: turning the piglet on its back, immobilising the piglet, catching and picking up the piglet, hitting the bottom of the piglet with the hand, shaking aluminium leaves near the piglet, shaking a plastic rattle paddle (Ukal, Canada Inc.) near the piglet or shooting a 6 mm plastic bullet on the back of the piglet with a spring gun (Taurus 24/7, Softair spring powered, San Francisco, USA). Each rough interaction was used at least once and proposed in a predetermined order. Contrary to GEN piglets, ROU piglets were not habituated to the trolley. As of the first day of training, piglets were pursued, caught and put roughly into the trolley by another unfamiliar handler. From four weeks post-weaning, piglets were pursued with a board (Ukal, Canada Inc.), pushed, hit with the hand and forced to climb the ramp into the trolley. The procedure was the same to get out of the trolley. By concern to the welfare of the piglets, all aversive treatments were intense enough to create some fear to the animal, but not to hurt them.

*Minimal contact* (MIN) aimed at avoiding contact with humans as much as possible. Piglets did not receive any human interaction or habituation to the trolley. As of the first day of training to the go/no-go task, the handler entered into the pen only to load on or unload piglets from the trolley. The handler remained calm and silent during the loading and quietly guided piglets to go into or out of the trolley by using a board.

Handlers involved in treatments and tests were three women and consistently apply a treatment and wore a black, white or striped black and white coverall and boots within a block of experiment in order to facilitate human discrimination by piglets. These coveralls differed from the blue or green coveralls worn by the usual piggery staff. Coveralls and roles were exchanged between the two blocks of experiment to standardise the association between treatments and coverall colour.

### Testing arena and apparatus design

**Fig 1:**
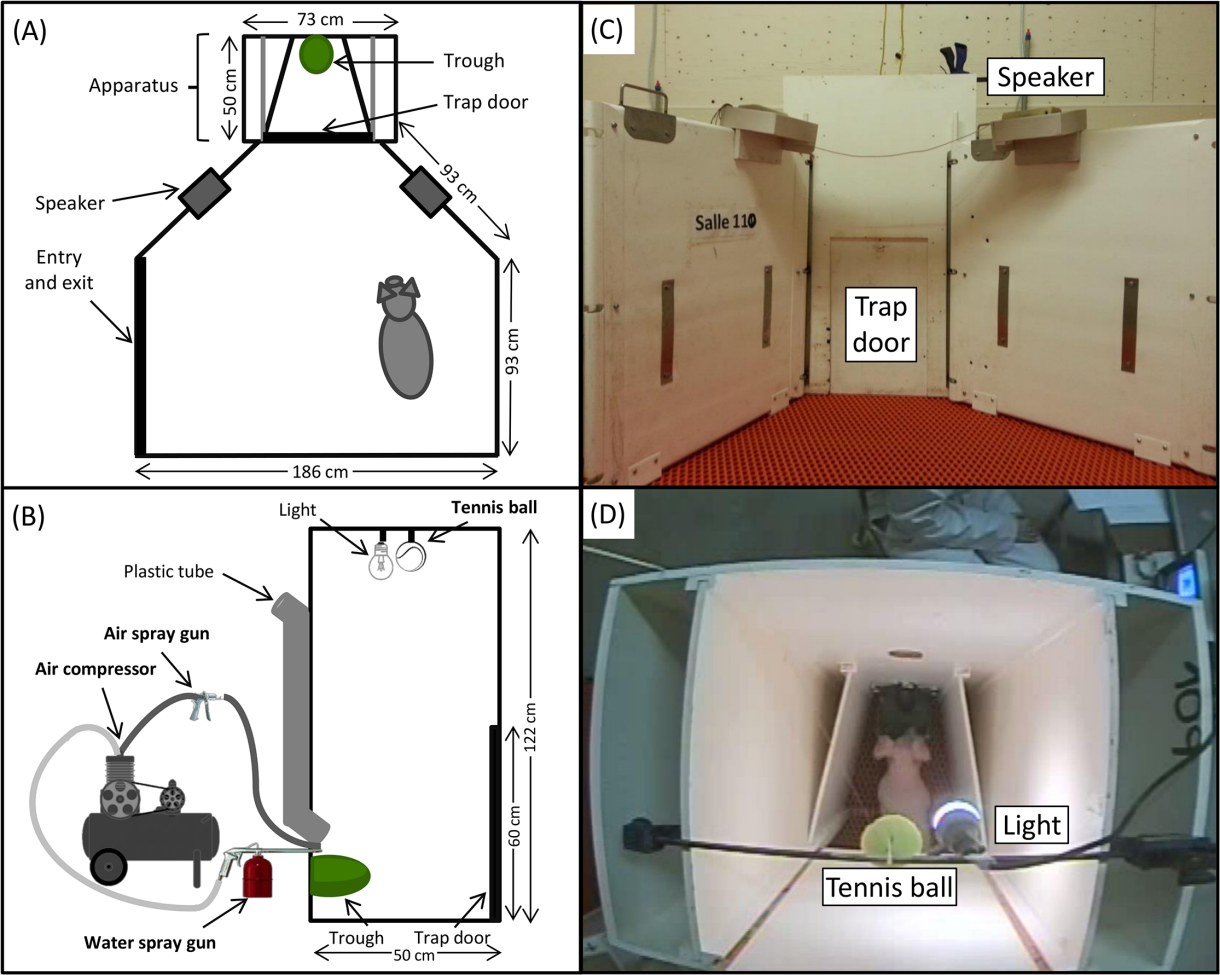
Overview of the testing arena and the apparatus. **(A)** Sketch of the testing arena viewed from the top, including the apparatus and the speakers; **(B)** Sketch of the apparatus viewed from the side, including the camera, the light, the plastic tube and the trough. In bold topography, a part of the material used for punishments, including the tennis ball relied on a fishing line, the air compressor relied on an air spray gun and a water spray gun; **(C)** Picture of the apparatus viewed from the front; **(D)** Picture of the apparatus viewed from the top with a piglet at the trough.

The testing arena was located in an experimental room separated from housing rooms and consisted of a 2.30 m^2^ arena made of plastic-coated expanded metal flooring with an apparatus on the narrow side (**Fig 1**). Two digital video cameras (Panasonic WV-CP 480, Panasonic, Mississauga, ON, Canada), one fixed above the apparatus, the other fixed above the testing arena, were used to record behaviour of piglets at 15 FPS and behaviour was analysed using a specialised recording and viewing software (Omnicast, Genetec Inc., Montréal, QC, Canada). The apparatus consisted of a wooden box (50.5 x 73 x 122 cm) opened at the top and fitted with a trap door (38 x 60 cm) that can be opened using a rope and a pulley system. The apparatus contained a black plastic tube opening onto a green porcelain trough. A light was fixed above the apparatus. A tennis ball was fixed at the top of the apparatus and linked up to a fishing line. A tube linked up to an air compressor and another one linked up to a water gun (itself linked up to the air compressor) opened onto the trough. Auditory cues were played using a computer linked up to two speakers positioned on each side of the testing arena. The testing arena was cleaned at the end of daily experiments.

### Testing procedure

#### Habituation

The method used to train piglets for the go/no-go task was adapted from a previous study in pigs [22]. Before habituation to the testing arena, piglets were previously habituated to food rewards (puffed wheat cereals, Sugar Crisps, Post, Niagara Falls, Canada) in their home pen. Then, two days before training, each group of three piglets was moved to the testing arena for two sessions of 12 min of habituation. Food rewards were left into the trough and new rewards were added each time piglets emptied the trough. The day before beginning of training, piglets were individually moved to the testing arena for two sessions of 8 min and received food rewards each time they reached the trough with the snout. Neither time delay limit nor auditory cues were applied during habituation and the trap door remained opened.

#### Training to the go/no-go task

Starting on the tenth day after weaning, piglets received two daily individual training sessions to the go/no-go task (one in the morning, one in the afternoon), five days per week. Piglets had to gradually learn discriminating two auditory cues, one positive (P cue) and one negative (N cue), varying in tonality and frequency in the available time. Auditory cues were computed using the software Reaper (module 4Front Piano, version 4.402). The high-pitched auditory cue was a C7 (2093 Hz) repeated 20 times over 4 seconds. The low-pitched auditory cue was a C1 (32.70 Hz) repeated 4 times over 4 seconds. The note was balanced between treatments; half of the piglets received the high-pitched auditory cue as P cue and the other half received the low-pitched auditory cue as P cue. Whereas the P cue predicted a reward, the N cue predicted a punishment given when the piglet reached the trough. Each training session was composed of six trials during which the auditory cue was played while the trap door was opening and again when the piglet ate the reward or received the punishment. The light into the apparatus was switched on for the trial and then switched off at the end of the trial to signal closing of the trap door. Piglets had a maximum of 35 seconds to reach the trough inside the apparatus with the snout before the trap door would be closed. Palatable food rewards (two pieces of cereals) were predictable and consistent whereas punishments varied across sessions. Each type of punishment was given three consecutive times and in the following order: a tennis ball fell on the back, a fresh water bowl spilled on the back, an air spray spouted in the face, a black plastic bag waved over [22], a fresh water spray spouted in the face. A correct response was defined as reaching the trough and eating the reward (i.e. a “go” response) following a positive cue and not reaching the trough (i.e. “no-go” response) following a negative cue.

Piglets were trained according to a decrease in the proportion of positive trials and an increase in the proportion of negative trials per training session across four levels. From level 2, piglets had to perform successfully for three training sessions, consecutive or not, to upgrade to next level.

- Level 1: The six trials were positive (6P). Piglets were trained to associate the positive auditory cue with the food reward delivered into the trough. When they successfully ate all of the six rewards within one training session, they upgraded to level 2.
- Level 2: Two neutral trials were incorporated (ratio of 4P/20). During neutral trials, no auditory cue was performed and no reward was given but the trap door was opened and the light switched on. This procedure ensured that piglets associate the reward with the auditory cue only. The criterion chosen to succeed a training session was a success at the four positive trials given.
- Level 3: Two negative trials were incorporated at a ratio of 4P/2N. The criterion chosen to succeed a training session was a success on at least 5 out of the 6 trials presented.
- Level 4: Half of the trials were positive, half were negative (ratio of 3P/3N). The criterion chosen to succeed a training session was a success on at least 5 out of the 6 trials.

The first trial of each training session was always positive but all other trials were pseudo-randomly proposed with never more than two similar cues followed. The experimenter was unfamiliar to the piglets and always hidden behind the apparatus. Thirty-two of the 54 piglets (59%) completed successfully the four levels of the training.

#### Cognitive Bias Test

Once piglets had completed the training to the go/no-go task, they were confronted to a series of cognitive bias tests (CBT). The response of piglets to new ambiguous auditory cues was recorded. Three levels of ambiguity were tested, namely an ambiguous cue 1/3 close to the P cue (AP cue, C3 = 130.81 Hz or C5 = 523.25 Hz, repeated 8 or 16 times, respectively), a median ambiguous cue 50/50 between P and N cues (AM cue, C4 = 261.63 Hz, repeated 12 times) and an ambiguous cue 1/3 close to the N cue (AN cue, C5 or C3, repeated 16 or 8 times, respectively). Approach responses to the trough following ambiguous cues were neither rewarded nor punished. The approach responses (Approaches (yes or not), latency to approach, percentage of time inside the apparatus and in contact with the trough) to trained and ambiguous cues were recorded and analysed. The judgement bias (or “risk taking”) was evaluated regarding the variables approach and latency to approach. In contrast, the variables of percentage of time spent inside the apparatus and in contact with the trough were not relied on judgement since piglets had already take their decision to approach, but rather illustrated exclusively the motivation and persistency to explore and/or seek for reward.

Whereas training sessions were performed without the handler, CBT were performed with or without the presence of a human observer (i.e. the handler who gave the treatment in the home pen for GEN and ROU piglets or unfamiliar observer for MIN piglets). Unfamiliar humans (men or women) wore blue or green coveralls and were stockmen from the piggery who were not involved in the management of piglets from the present project. In addition, they never participated to a CBT more than once for a same piglet to ensure that they were really unfamiliar for piglets. The order of sessions of CBT with or without the observer (period with the observer: present/absent or absent/present) was controlled and balanced between groups and treatments. The observer was outside but close to the testing arena so piglets could see the top part of his body and his face. He remained motionless with his arms along the body and gazed at piglets. Piglets received twice each ambiguous cue with and without the observer presence (i.e. two repetitions of each ambiguous cue with the human observer and two without him). Ambiguous trials were divided between at least four CBT sessions and were played after successful P-N or N-P pair of trials. CBT sessions in which piglets immediately failed trained cues and did not receive any ambiguous cues were removed from the subsequent analyses since it was considered that piglets were not enough concentrated or motivated during these sessions. The last trained cue (P or N), played before ambiguous cues, was balanced between trials since it can influence the interpretation of the ambiguous cue. Up to three ambiguous trials were played within one CBT session. If piglets did more than two errors on learnt cues, the CBT session ended. Thus, a maximum number of 11 trials were performed per CBT session.

### Statistical analyses

The SAS software (version 9.2; SAS institute Inc.) was used to analyse data and the significance threshold was 0.05. All data were tested to see if they satisfied requirements for parametric testing.

Treatment effect on success in the task learning (yes or no) was analysed with a logistic model. In addition, the influence of the treatment and the presence of a human observer on the number of CBT sessions needed to play all ambiguous cues was evaluated using ANOVA for mixed models with treatment (three levels, GEN, ROU or MIN, respectively), observer (two levels, yes or no, respectively), and their interaction as fixed effects. These results are presented as least square-means ± SEM in the text.

Other data were analysed following the recommendations of Gygax [27]. Approaches (yes or no) were analysed using logistic regression with a binomial distribution. Latency to approach was analysed using ANOVA for mixed models, only for trials in which piglets approached. Percentages of time spent inside the apparatus and in contact with the trough were analysed in the same way, only for trials in which piglets approached, after performing an angular transformation. The experimental unit was the trial but the hierarchical levels of group, piglet and CBT session were incorporated in the random effect and the piglet was specified as the subject to correctly assign available degrees of freedom to the fixed effects. In addition, the degree of freedom was corrected with the Kenward-Roger adjustment. For the analysis of the discrimination of trained P and N cues during the last three training sessions, terms treated as fixed effects were cue (two levels, P or N, respectively), treatment and their interaction. Terms treated as fixed effects during CBT were cue (five levels, P, AP, AM, AN or N, respectively), treatment, observer and their interactions. In addition, the effect of repeating ambiguous cues was evaluated by using the same models, but only with ambiguous cues, and by adding repetition (two levels, 1 or 2, respectively) and respective interactions as fixed effects. Data from analyses are presented as least square-means ± SEM in the text. Adjusted means and confidence intervals for percentage of time were back-transformed to the original scale for presentation in figures.

## Results

### Task learning

The proportion of subjects that completed the training did not differ significantly between treatments (N_GEN_ = 9 (50%), N_MIN_ = 12 (67%), N_ROU_ = 11 (61%), $\chi_{2}^{2}$ = 1.06, *P* = 0.59). Piglets that completed the task learning successfully discriminated both positive and negative cues during the three last training sessions (approaches: 74.8 ± 5.9% *vs* 15.8 ± 4.3%, for positive and negative cues respectively, *F*_1,559.9_ = 157.86, *P* < 0.0001), independently of the treatment (GEN: 40.8 ± 11.9%, ROU: 42.6 ± 11.3%, MIN: 44.8 ± 11.1%, *F*_2,14.47_ = 0.03, *P* = 0.97).

### Cognitive Bias Test

Three animals stopped approaching the trough during CBT and therefore were excluded from the analysis, leaving a sample size of 29 piglets (N_GEN_ = 8, N_MIN_ = 11, N_ROU_ = 10). Since ambiguous cues were played following a successful P-N or N-P pair of trials and piglets failed to some trained cues, the number of CBT sessions required to play all the ambiguous cues varied between piglets from 4 to 15 sessions. Analyses revealed that repeating ambiguous cues did not influence the percentage of approach of piglets (-0.69 ± 0.30 and -0.41 ± 0.28, repetition 1 and 2 respectively, *F*_1,298_ = 0.61, *P* = 0.54), the latency to approach (11.3 ± 2.2 and 7.9 ± 2.0, repetition 1 and 2 respectively, *F*_1,77.6_ = 1.75, *P* = 0.18) or the percentage of time spent inside the apparatus (0.32 ± 0.04 and 0.33 ± 0.04, repetition 1 and 2 respectively, *F*_1,276_ = 0.06, *P* = 0.80) and in contact with the trough (0.19 ± 0.03 and 0.21 ± 0.03, repetition 1 and 2 respectively, *F*_1,243_ = 0.29, *P* = 0.59).

#### Judgement bias

**Fig 2:**
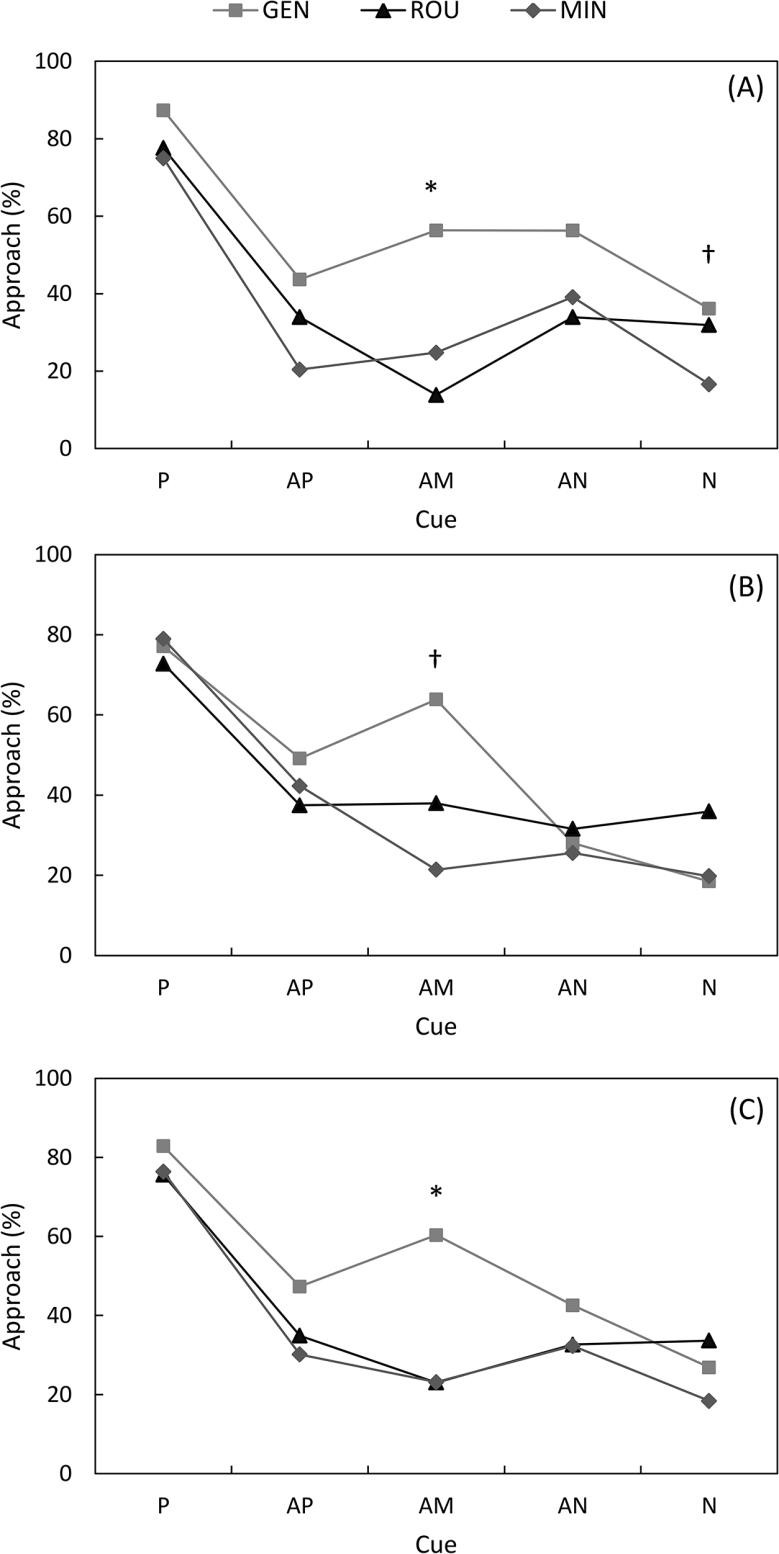
The nature of the experience with the handler biases the judgement of piglets towards AM cues. Average proportion of approach (i.e. “go” response) in response to the five cues during cognitive bias tests **(A)** in which the human observer was absent; **(B)** in which the human observer was present; **(C)** or the combination of the two conditions, for piglets from gentle (GEN, light grey squares), rough (ROU, black triangles) and minimal contact (MIN, dark grey diamonds) treatments (back-transformed least square means; P, trained positive cue; AP, ambiguous cue nearest positive cue; AM, ambiguous median cue; AN, ambiguous cue nearest negative cue; N, trained negative cue) († *P* < 0.10; * *P* < 0.05).

The number of CBT sessions required to play all the ambiguous cues was not influenced by treatment (GEN: 4.5 ± 0.5 sessions; ROU: 4.3 ± 0.5 sessions; MIN: 3.4 ± 0.4 sessions, per set of sessions with or without an observer, *F*_2,26_ = 1.63, *P* = 0.21). The main effects on behavioural variables following each cue are summarised in **Table 1**. The results show that the interaction between cue and treatment had an impact on behavioural responses of piglets during CBT. In addition, the interaction between observer and treatment tended to have an impact on the proportion of approach. **Fig 2A and 2B**describe the mean percentage of approach to the trough of piglets from each treatment for both trained and ambiguous cues during CBT in absence or presence of the human observer. Analyses showed that differences between treatments in the proportion of approach, regardless of the cue, were significant in the absence of the human observer (*F*_2,21.37_ = 3.32, *P* = 0.05). Specifically, GEN piglets approached more often following playback of the AM cues than ROU piglets when the observer was absent (0.25 ± 0.56 vs -1.83 ± 0.67, *t*_263.3_ = -2.40, *P* = 0.04), whereas MIN piglets were intermediate (-1.11 ± 0.54) and tended to differ from GEN (*t*_198.8_ = -1.76, *P* = 0.08) and did not significantly differ or even tend to differ from ROU (*t*_237.4_ = 0.84, *P* = 0.40) piglets (**Fig 2A**). Although there were no overall treatment effect in presence of the human observer (*F*_2,12.89_ = 0.31, *P* = 0.74), an effect of the interaction between treatment and cue was observed (*F*_8,748.8_ = 2.00, *P* = 0.04). Indeed, when the handler was present, GEN piglets tended to approach more often following playback of AM cues than MIN piglets (0.57 ± 0.64 vs -1.30 ± 0.61, *t*_90.72_ = -2.15, *P* = 0.08), whereas ROU piglets were intermediate (-0.49 ± 0.59) and did not significantly differ from GEN (*t*_79.7_ = -1.23, *P* = 0.43) and MIN piglets (*t*_63.47_ = -0.97, *P* = 0.59) (**Fig 2B**).

**Table 1:** Main effects on approach behaviour following playback of trained (positive (P) and negative (N)) and ambiguous (AP, AM and AN) cues during cognitive bias tests, in presence or absence of a human observer, for piglets having previously experienced gentle (GEN), rough (ROU) and minimal contact (MIN).

Variables		Cue	Treatment	Observer	Cue x Treatment	Cue x Observer	Observer x Treatment	Cue x Observer x Treatment
Proportion of approach	*F*	67.00	1.59	0.14	2.20	1.88	2.89	0.66
	*P* ^1^	**<0.0001**	ns	ns	**0.02**	ns	***0*.*06***	ns
Latency to approach	*F*	21.94	0.01	0.50	0.72	0.35	0.52	0.53
	*P* ^1^	**<0.0001**	ns	ns	ns	ns	ns	ns
Percentage of time inside the apparatus	*F*	100.92	0.43	5.90	2.41	1.67	2.24	0.71
	*P* ^1^	**<0.0001**	ns	**0.02**	**0.02**	ns	ns	ns
Percentage of time	*F*	321.28	0.65	4.14	2.78	2.05	1.53	0.55
in contact with the trough	*P* ^1^	**<0.0001**	ns	**0.04**	**0.007**	***0*.*09***	ns	ns

^1^: ns: *P* > 0.10.

Nonetheless, because the effect of the interaction between observer and treatment was only a tendency, data were also pooled for a global analysis. Again, the proportion of approach was influenced by treatments following playback of AM cues (*F*_2,79.97_ = 4.14, *P* = 0.01, **Fig 2C**). Specifically, GEN piglets approached more often following playback of AM cues than ROU (0.42 ± 0.46 vs -1.21 ± 0.48, *t*_87.37_ = -2.44, *P* = 0.03) and MIN (-1.20 ± 0.44, *t*_81.15_ = -2.54, *P* = 0.02) piglets, whereas ROU and MIN did not differ (*t*_73.08_ = 0.01, *P* = 1.00) (**Fig 2C**). However, the proportion of approach was not influenced by treatments neither following playbacks of trained P and N cues (P cues: 1.57 ± 0.36, 1.13 ± 0.32 and 1.17 ± 0.31, respectively for GEN, ROU and MIN piglets, *F*_2,20.15_ = 0.52, *P* = 0.60; N cues: -1.00 ± 0.34, -0.67 ± 0.31 and -1.49 ± 0.32, respectively for GEN, ROU and MIN piglets, *F*_2,17.76_ = 1.72, *P* = 0.21) nor following playbacks of AP or AN cues (AP cues: -0.11 ± 0.46, -0.62 ± 0.44 and -0.84 ± 0.43, respectively for GEN, ROU and MIN piglets, *F*_2,67.24_ = 0.71, *P* = 0.49; AN cues: -0.30 ± 0.47, -0.72 ± 0.44 and -0.74 ± 0.42, respectively for GEN, ROU and MIN piglets, *F*_2,67.22_ = 0.31, *P* = 0.74) (**Fig 2C**).

**Fig 3:**
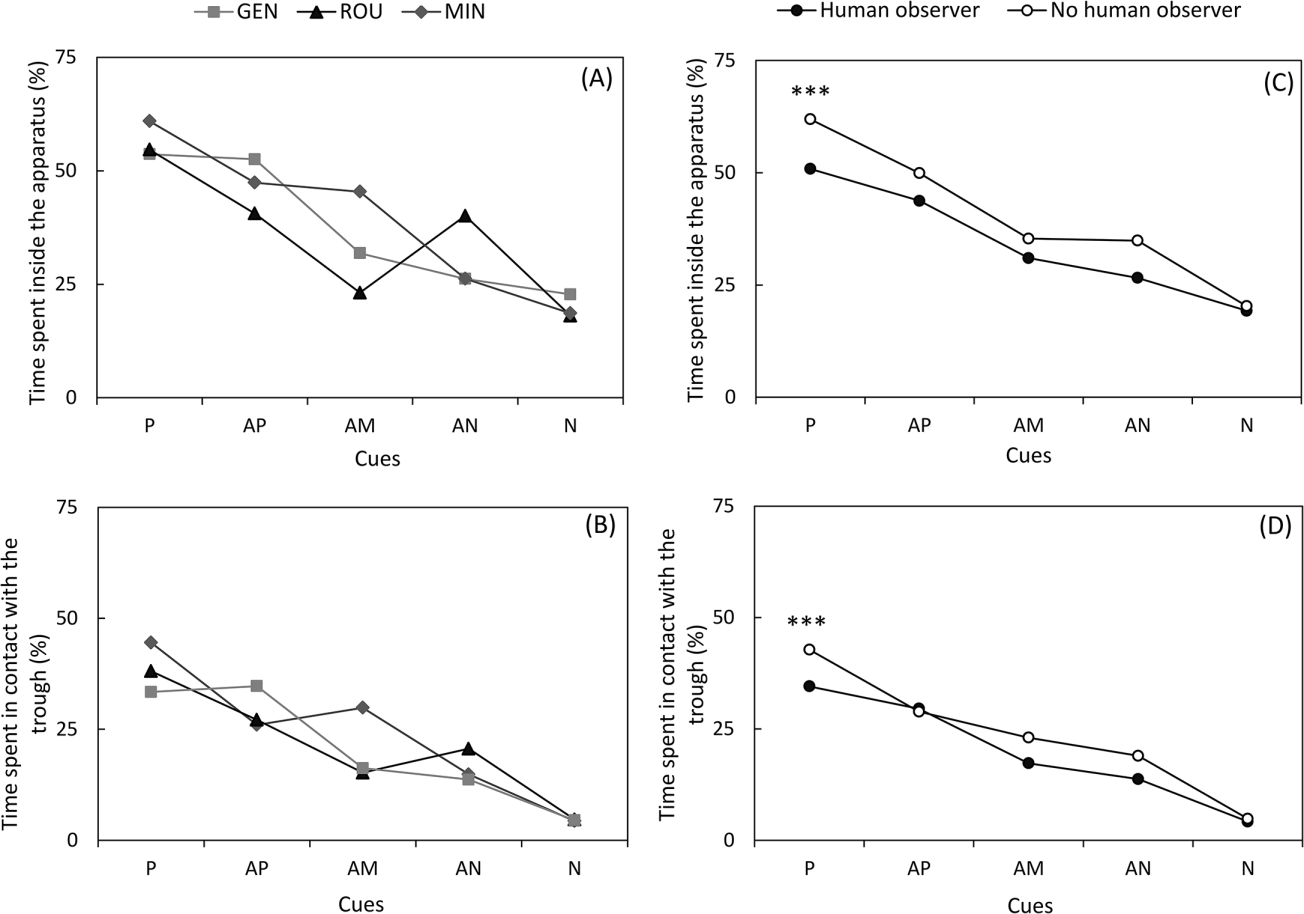
Percentages of time spent inside the apparatus and in contact with the trough are affected by the presence of a human observer, and to a lesser extent, by treatments. **(A)** Average percentage of time spent inside the apparatus and; **(B)** Average percentage of time spent in physical contact with the trough following playbacks of the five cues during cognitive bias tests for piglets from gentle (GEN, light grey squares), rough (ROU, black triangles) and minimal contact (MIN, dark grey diamonds) treatments, regardless of the presence or not of the handler; **(C)** Average percentage of time spent inside the apparatus and; **(D)** Average percentage of time spent in physical contact with the trough following playbacks of the five cues during cognitive bias tests in the absence (white circles) or presence (black circles) of the human observer, regardless of the treatment. (back-transformed least square means; P, positive cue; AP, ambiguous cue nearest positive cue; AM, ambiguous median cue; AN, ambiguous cue nearest negative cue; N, negative cue) (*** *P* < 0.0001).

The **Table 1**shows an effect of the interaction between cue and treatment on the percentage of time spent inside the apparatus and in contact with the trough, meaning that changes in the approach behaviour of piglets from P to N cues progressed differently according to the treatment (**Fig 3A and 3B**). Indeed, the decrease of the approach behaviour from positive to negative cues of GEN piglets seems to be less pronounced than for MIN piglets. However, the analysis for each cue separately did not show any treatment effect.

#### Presence of the human observer

The performance of piglets was lower when the observer was present since the number of CBT sessions required to play all the ambiguous cues was higher with than without the observer (4.6 ± 0.3 vs. 3.6 ± 0.3 sessions, respectively, *F*_1,26_ = 5.18, *P* = 0.03), suggesting that piglets failed more often following P and/or N cues when the observer was present. Results show that the presence of a human observer during CBT influenced the percentage of time spent inside the apparatus and in contact with the trough (**Table 1**). Overall, piglets spent less time inside the apparatus and in contact with the trough when the handler was present regardless of the cue (time inside the apparatus: 0.68 ± 0.02 vs 0.78 ± 0.02, *F*_1,669_ = 18.40, *P* < 0.0001; time in contact with the trough: 0.54 ± 0.02 vs 0.62 ± 0.02, *F*_1,618_ = 16.89, *P* < 0.0001; **Fig 3C and 3D**). When considering cues separately, the effect was significant for P cues only (time inside the apparatus: 0.79 ± 0.03 vs 0.91 ± 0.03, *F*_1,402_ = 20.05, *P* < 0.0001; time in contact with the trough: 0.63 ± 0.03 vs 0.71 ± 0.03, *F*_1,406_ = 16.51, *P* < 0.0001, **Fig 3C and 3D**). In addition, as mentioned above, the interaction between observer and treatment tended to modulate the proportion of approach (**Table 1**).

## Discussion

The nature of the chronic experience with the handler induced a judgement bias confirming the hypothesis that the way humans behave with animals can modulate the emotional state of the animal and hence its welfare. In addition, the presence of a human observer (i.e. the familiar handler for GEN and ROU piglets and unfamiliar humans for MIN piglets) during cognitive bias tests disturbed piglets, especially for GEN piglets.

Piglets which received gentle contact over a long period of time were more likely to approach the trough in response to the ambiguous median auditory cue than piglets which received rough contact, piglets from minimal contact being intermediate. This decision-making suggests that piglets from gentle contact treatment expected the outcome to the ambiguous cues more likely to be the same as with the positive cue (i.e. optimistic bias) and thus, enhanced risk taking compared to piglets from rough contact which chose to favor caution (i.e. pessimistic bias). The emotional state, at any given time, biases information processing [3] and influences how organisms perceive situations or stimuli. With experience, piglets handled gently may not only form a positive memory of humans and be more willing to approach or be approached by gentle or unfamiliar humans [16, 17], but also perceive less negatively and be less wary and fearful under uncertain situations than piglets handled roughly. These results corroborate with previous studies on human-pig relationship [28, 29] that originally showed the impact of handling with different valences on pig behaviour and welfare. In pigs, aversive experience with humans involving electric shocks is susceptible to induce a chronic stress response, which translates in higher free corticosteroid concentrations even in the absence of negative handlers. This leads to lower growth rate, impairment of reproductive functions, and fear of humans [28, 29]. In the present study, negative treatments were clearly less aversive than electric shocks but they were sufficient to have an impact on emotional states. Hence the impact of humans on the emotional state of the piglets as observed in the present study gives a coherent picture about the involvement of humans in animal welfare. Contrary to the present study, it seems that in some contexts, release from stressful situations may also lead to optimistic biases [13, 30, 31]. For instance, shearing, a routine husbandry procedure in sheep in which humans are involved, provokes an acute stress response and is accompanied by elevated plasma cortisol concentrations [32]. Although the link between this procedure and the perception of humans is poorly understood, it has been demonstrated that sheep exhibit positive judgement bias following release from shearing [30]. Other examples in which stressful situations induced positive judgement bias have been shown, including 6h/day restraints for three consecutive days before cognitive bias tests in sheep [31] or five day training using negative reinforcements in horses [13]. All these treatments were novel and applied on a relatively short period of time and the authors supposed that stressed animals may have sought something positive during CBT to counteract their negative experience. In contrast, the present study involved a long lasting experience, including unpredictable and sudden interventions inside the home pen (2/week) and daily rough moving between pens and testing arena, which was incorporated into the daily life of animals. Hence, the situation was deeply different and piglets roughly handled may have shown free-floating negative mood throughout the experiment. However, no judgement bias was observed for the ambiguous cues nearest positive cue and nearest negative cue. The paradigm supposes that a negative bias in response to an ambiguous cue closest to the positive cue may reflect a decreased expectation of positive event, a symptom of depression, whereas a negative bias in response to an ambiguous cue closest to the negative cue may indicate an increased expectation of negative events, a symptom of anxiety [4]. Using different probe degrees may have potentially provided not only an indicator of emotional states with different valences (e.g. hopeful vs hopeless), but also an indicator of emotional states with comparable valences but different degrees of arousal (e.g. anxiety vs depression), as observed in previous studies [33, 34]. The absence of treatment effect prevented any conclusion about the potential arousal level (anxiety vs depression) of piglets.

It is particularly noteworthy to see how the presence of a human observer (i.e. the familiar gentle or rough handler for GEN and ROU piglets, respectively, or an unfamiliar handler for MIN piglets) disturbed piglets during cognitive bias tests. Although piglets maintained their performance in discriminating cues, they spent less time inside the apparatus and in contact with the trough when the human observer was present. It was originally shown that in absence of humans, isolated young pigs which have received minimal interactions with humans prefer access to sight and relative proximity with conspecifics as well as comfortable lying surface instead of a mirror [35]. However when a human is present, their preferences for comfort switch for social enrichments including mirror or conspecifics. Activities such as rest on a mat or exploration of objects (e.g. the apparatus in the present study) provide comfort or pleasure but in situations of perceived threat (unfamiliarity is potentially threatening, [36]), social animals may shift their activity to focusing their attention towards threat and/or seeking for social support. In the present study, the presence of an observer was unfamiliar to piglets from all treatments since they were always trained without having a human in sight. It seems that they were more anxious, or at least attentive towards the observer, and they were less willing to explore or seek for rewards than when the observer was absent. This may be explained by the loss of visual control over the observer when being in the apparatus. Roughly handled piglets should have been particularly wary and refuse to participate to CBT when the familiar rough handler was present but it was not the case. Perhaps the value of food rewards was higher than the risk to lose sight of the familiar rough handler for few seconds. In addition, the way an animal perceive a same human being may vary according to the context. This new context, in which the negative handler was motionless outside of the pen may have induced a lower emotional response. Nevertheless, regardless of the treatment, the presence of the human observer did not bias the judgement of piglets since the way they interpreted the ambiguous situation, as observed by the choice of approaching (or not) and the latency to approach ambiguous cues, was not influenced by the presence of the handler. This does not preclude that differences between treatments on the proportion of approach tended to appear more clear-cut in absence of the observer, reinforcing the idea that the presence of an observer, an unfamiliar situation for piglets, disturbed them.

Some methodological aspects should also be discussed. Firstly, the present study demonstrates that piglets can be trained to discriminate auditory cues that differ quantitatively. In the study of Douglas et al. [22], pigs failed to discriminate notes differing only in frequency (Hz). In pigs, the various grunts and calls do not appear to have specific meanings, but the intensity, the frequency and the duration vary with the type of situations [37, 38]. Thus, it has been supposed that pigs may discriminate more easily notes differing in frequencies (Hz) but also in repetition (number) in the available time. Since high tonalities and longer sound durations are associated with experiences considered highly stressful in pigs [38], played cues could have a kind of meaning for pigs and influence their behavioural responses. Therefore, notes associated with positive and negative cues were balanced between treatments. Secondly, to optimise learning, pre-experiments showed that it was important to find a way to maintain a strong motivation to approach during positive trials and a strong motivation to avoid approaching during negative trials over time. Whereas some authors rationed pigs for cognitive bias experiments [22, 25], others provided food *ad libitum* [24]. Rationing piglets proved to be efficient to maintain such level of motivation, even if it seems that it also motivated them to take risks (incorrect responses to negative cues). In addition, punishments used in negative trials were regularly changed to prevent from habituation. Training duration was adapted according to individual learning performances. It is supposed that this individual learning may have decrease inter-individual differences of approaches to learnt P and N cues during CBT. A potential criticism of the present study is about the low success rate in the task learning. Overall, 59% of piglets learnt the task, which is poor compared to other studies in which all pigs learnt the task [22, 23, 25]. Compared to other studies in pigs, animals used in the present study were younger (i.e. four weeks old) when they started training. Perhaps discriminative learning is much more difficult for weaned piglets than for older pigs. One exception is the study from Düpjan et al. [24] who worked with 6–9 weeks old pigs, with two pigs out of 17 that failed to learn a discriminative spatial task. In another study [39], a go/no-go task with young pigs (i.e. six weeks old) comparable to the one used in the present study was also learnt with a success rate of 50% within 25 sessions of 12 trials. In the same study, a significantly higher success rate (i.e. 100% within 16 sessions of 13 trials) was observed with five months pigs by using an active-choice task. Although these older animals were already used in other cognitive experiments [40], authors suggested that active-choice tasks, for which responses are operant for the two trained cues (e.g. animals have to go-left to positive cues and have to go-right to negative cues), gave clearer and more consistent results than go/no-go tasks, especially because they allow differentiating a pessimistic response (approach of negative location/lever) from an omission.

### Conclusion

The present study shows that weaned piglets are endowed with cognitive abilities to learn a go/no-go task, just as older pigs. This study originally demonstrates that the emotional state of a farm animal such as piglets can be affected by the way humans interact with them. Gentle interactions with humans induced more positive emotional states, as observed by the optimistic judgement bias of gently handled piglets compared to piglets which were roughly handled or received minimal contact. In addition, the presence of a human as an observer during cognitive bias tests caught the attention of piglets and affected their motivation to explore the apparatus and the trough, even though it did not significantly bias their judgement. The demonstration of such cognitive and emotional abilities in farm animals should be taken into account in animal welfare research and management. Furthermore, it should modify our own perception of animals and may lead to the development of techniques by which humans can, not only decrease bad mood, but also trigger positive emotional states of animals.

## Supporting Information

S1 File Datasets used for the statistical analyses. (XLSX).
(XLSX)
